# DARC and Anti-Duffy Antibodies in the Line of Fire: The Challenges in Pinpointing the Etiology of Microcirculation Inflammation to a Distinct Entity

**DOI:** 10.3389/ti.2025.15601

**Published:** 2026-01-12

**Authors:** Farsad Eskandary, Günther F. Körmöczi, Marlies Schönbacher, Gottfried Fischer, Ingrid Faé, Sabine Wenda, Daniela Koren, Rainer Oberbauer, Roman Reindl-Schwaighofer, Andreas Heinzel, Johannes Kläger, Nicolas Kozakowski, Stephan Segerer, Konstantin Doberer, Luis G. Hidalgo, Helga Schachner, Georg A. Böhmig, Heinz Regele

**Affiliations:** 1 Division of Nephrology and Dialysis, Department of Medicine III, Medical University of Vienna, Vienna, Austria; 2 Department of Blood Group Serology and Cell Therapy, Medical University of Vienna, Vienna, Austria; 3 Department of Pathology, Medical University of Vienna, Vienna, Austria; 4 Division of Nephrology, Dialysis and Transplantation, Kantonsspital Aarau, Aarau, Switzerland; 5 Histocompatibility and Immunogenetics Laboratory, University of Alabama at Birmingham, Birmingham, AL, United States

**Keywords:** antibody-mediated rejection (AMR), humoral rejection, DSA, donor specific antibodies, Duffy blood group

## Abstract

Antibody-mediated rejection (ABMR) due to non-HLA alloantibodies has gained substantial attention in transplantation research. One candidate for such non-HLA reactivity is the Duffy blood group carrier molecule DARC, which is not only expressed on erythrocytes, but also on kidney microvascular endothelial cells and is postulated as a potential transplantation-relevant alloantigen. However, *in vivo* observation of anti-Duffy antibodies as trigger of microvascular inflammation (MVI) is lacking. Here we propose a direct relationship between preformed anti-Duffy (anti-Fy^a^) antibodies, complement deposition (C4d) in peritubular capillaries (PTC), and MVI. Double immunofluorescence for DARC and C4d in sequential biopsies revealed a striking overlap of DARC expression and C4d staining that was completely restricted to the peritubular capillaries. Remarkably, MVI was confined to PTC with complete absence of glomerulitis and lack of preformed anti-HLA DSA. Retrospective analysis revealed a self-limiting posttransplant flare of a low-level anti-DQ8 DSA after blood transfusions and a high missing-self KIR ligand constellation. Concomitant occurrence of non-HLA and anti-HLA reactivities next to missing-self constellations substantially complicates the assessment of individual contributions for the development and propagation of MVI. Due to the strictly confined distribution of DARC to PTC our report provides *in vivo* evidence that anti-Fy^a^ alloantibodies may associate with MVI.

## Graphical Abstract

**Figure:**
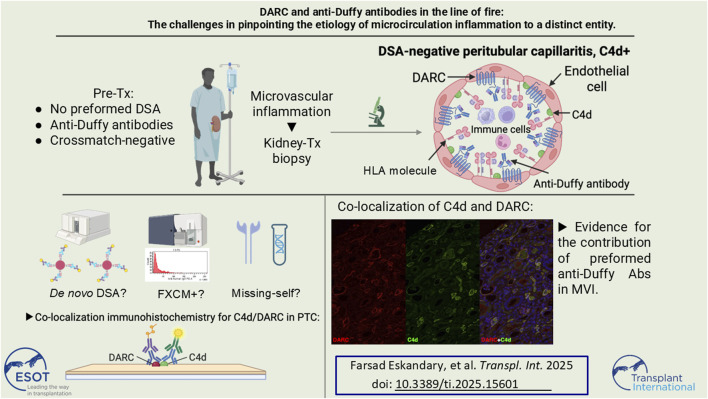

## Introduction

Antibody-mediated rejection (ABMR) constitutes one of the major causes for late allograft loss after kidney transplantation [1, 2]. While the majority of ABMR cases is positive for anti-HLA donor-specific antibodies (DSA), some evidence also supports a role of non-HLA antigens as primary targets of alloantibodies [3]. Clinicians usually only search for such antibodies, when serologic evidence of anti-HLA DSA is lacking despite typical ABMR lesions in biopsies. Detection of non-HLA alloantibodies is challenging, as only a limited number of validated assays are commercially available. Furthermore, the most comprehensive Luminex-based non-HLA antibody assays failed to correlate in a substantial proportion, hindering the development of recommendations for their routine testing [4, 5]. The occurrence of ABMR lesions in anti-HLA DSA-negative patients has recently been introduced as a diagnostic category of the Banff classification termed “microvascular inflammation (MVI) DSA and C4d negative,” because some studies observed its detrimental impact on graft survival [6, 7]. In this context it is of interest that particular donor/recipient HLA class I mismatches may trigger NK cells via KIR’s through the ‘missing-self’ pathway, leading to MVI through antibody-independent mechanisms [8].

The glycoprotein “Duffy antigen receptor for chemokines” (DARC) encoded on chromosome 1 constitutes a transfusion-relevant blood group system, belongs to the family of seven-transmembrane proteins, and exhibits clinically relevant Duffy blood group antigens (Fy^a^ or Fy^b^, depending on genotype) [9]. Its expression in the kidney vasculature is almost exclusively limited to the endothelium of peritubular capillaries (PTC) and postcapillary venules [10]. DARC was shown to be upregulated during kidney transplant rejection processes and its expression correlated with the extent of interstitial fibrosis in biopsies showing ABMR [11, 12]. Also, in gene expression profiling of kidney transplant biopsies [Molecular Microscope Diagnostic System (MMDx™) and NanoString®], DARC appeared as one of the most prominent ABMR-associated transcripts [13, 14]. Apart from a possible role in rejection, DARC has also been proposed as a transplantation-relevant antigen due to the fact that Duffy alloantibodies can form after sensitizing events [15, 16]. DARC genotype-mismatch constellations in kidney transplantation showed varying associations with respect to the occurrence of rejection, enhanced fibrosis and reduced graft survival. However, *in vivo* proof of such antibodies to directly contribute to ABMR are lacking [17–19].

Here we present the case of a patient with preformed anti-Fy^a^, who received a kidney transplant from a Fy(a+b-) donor and subsequently developed early C4d+ microvascular inflammation (MVI), at that time without the evidence of any anti-HLA DSA in serum. We retrospectively performed double immunofluorescence for C4d and DARC in biopsies to assess a potential co-localization of the antigens and their spatial relation to histologic signs of rejection. In this paper we describe the process of corroborating our findings, thorough retrospective workup of patient sera revealed the complexity of the observed MVI with respect to a causative role of anti-Fy^a^ antibodies.

## Methods

### Biopsies

All biopsies were graded according to the 2022 update of the Banff classification. For C4d immunohistochemical and immunofluorescence staining we used a polyclonal anti-C4d antibody (BI-RC4D; Biomedica, Vienna, Austria) and for DARC immunofluorescence we used a mouse monoclonal anti-human DARC-Fy6 antibody (a generous gift from the laboratory of Prof. Yves Colin, INSERM). The detailed protocol of our DARC and C4d double-immunofluorescence is provided in the Supplementary Appendix.

### Anti-Duffy Antibody Titration

Anti-Fy^a^ titration was performed using indirect antihuman globulin technique in gel matrix (MicroTyping system, Bio-Rad, Vienna, Austria).

### Anti-HLA Antibody Assessment, HLA and KIR Typing

HLA antibody detection was performed at our ISO-certified HLA laboratory, using LABscreen single-antigen flow-bead assays (One Lambda, Canoga Park, CA). Details with respect to donor/recipient high-resolution HLA typing, anti-HLA reactivities, HLA eplet mismatch, KIR typing and missing-self and are provided in the Supplementary Appendix.

### FXCM Crossmatch

A detailed technical description of the FCXM is provided in the Supplementary Appendix. In brief, mononuclear cells from donor spleen were extracted using LymphoprepTM density medium. After pronase digestion to deplete unspecific binding, sera were incubated with B and T lymphocytes and incubated with corresponding antibody-mixes. Controls were performed to account for unspecific binding, enzymatic digestion and equal HLA distribution on cell surfaces. Positivity threshold for FXCM results was >6000 MFI above the mean of negative control values.

## Results

In 2017, a 45-year-old male on peritoneal dialysis with a history of opioid abuse and hepatitis C-associated membranoproliferative glomerulonephritis (nucleic acid test-negative since 2015), received his first ABO-compatible deceased donor kidney transplant from a 51-year-old male. HLA mismatch was 0/1/1/1/1/1 (A/B/C/DR/DQ/DP), pretransplant CDCXM was negative and single antigen bead (SAB) testing revealed no preformed anti-HLA DSA (latest vPRA for HLA class I and II: 0%, highest historic vPRA: 7%). Anti-IL2 receptor antibody basiliximab was administered on days 0 (d0) and d4 (20 mg each) and maintenance immunosuppression consisted of standard triple immunosuppression with tacrolimus, mycophenolate-mofetil and steroids.

Upon transplant offer, previously identified donor-specific anti-Fy^a^ IgG was detectable, with an anti-Fy^a^ antibody titer of 64 (donor and recipient *DARC* genotypes FY*A and FY*B, respectively). Anti-Fy^a^ immunization was most likely due to prior RBC transfusion because of an episode of idiopathic autoimmune hemolytic anemia in 2005. We decided against any desensitization scheme, but to liberally perform transplant biopsies in case of graft dysfunction. The post-transplantation course was complicated by delayed graft function (DGF) most likely due to peri-renal hematomas that impaired global allograft perfusion, requiring two surgical revisions within the first week, and hemodialysis on day four (d4). A concomitant allograft biopsy on d4 contained very little cortical tissue, but showed severe tubular injury with tubular necrosis without signs of rejection and was C4d-negative. A second biopsy on d14 showed resolving tubular injury without TCMR, but now diffuse peritubular capillaritis (ptc2) and minimal linear C4d in PTC (C4d1), suggestive of antibody-mediated injury. Anti-HLA DSA were not detected. Meanwhile, the anti-Fy^a^ antibody titer had decreased to just above the detection level. Due to this early complicated course, with serum creatinine values stabilizing at around 1.5 mg/dL and absence of albuminuria/proteinuria, no anti-rejection treatment was initiated. At month three serum creatinine increased to >2 mg/dL, which prompted us to administer a steroid bolus before performing a biopsy, that still showed diffuse ptc2, but this time also a strong linear C4d-positivity in PTC (C4d2). There was still no sign of TCMR, but surprisingly we found mild diffuse interstitial fibrosis (ci3, affecting 70% of cortex) without chronic tubular damage (ct0); in electron microscopy no doubling of PTC basement membranes was found (MLPTC0). Repeated testing confirmed the absence of anti-HLA DSA, an anti-Fy^a^ titer decreasing below the detection limit and serum creatinine remaining at around 2 mg/dL. We decided to not further intensify immunosuppression, as our patient appeared prone to infectious complications (paralytic ileus with sepsis 4 months and pneumonia with subsequent right-sided surgical decortication for empyema 7 months post transplantation). Tacrolimus trough-level goal was set between 4 and 8 ng/mL and was mostly achieved with singular outliers in the upper and lower ranges (Median tacrolimus level day 14 until month ten after transplantation: 5.9 ng/mL, IQR: 4.3–6.4 ng/mL). Mycophenolate-mofetil was switched to azathioprine at month two because of gastrointestinal side effects. Torque-Teno-Virus PCR was assessed as a measure for global immunosuppression and was in the range of 10^4^ and 10^8^ copies, indicating no excessive immunosuppressive effect.

**FIGURE 1:**
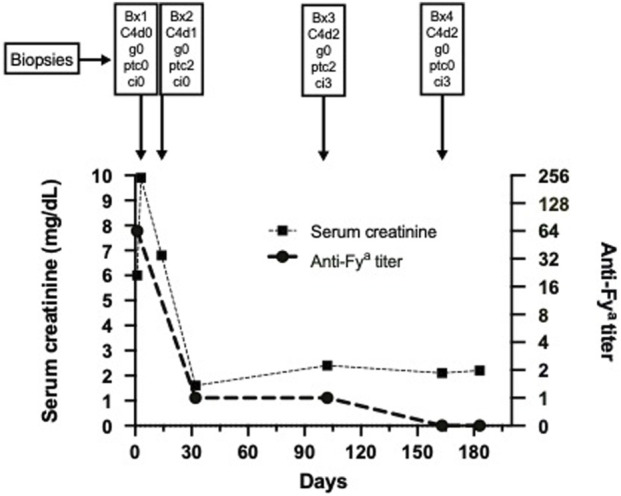
Serum creatinine course and anti-Fy^a^ antibody titer over time together with corresponding biopsy results. Abbreviations: Bx, biopsy; g, glomerulitis; ptc, peritubular capillaritis; ci, chronic interstitial fibrosis; C4d, complement split product C4d.

At month five BK-viremia (max: 6.5 × 10^3^ copies/mL) together with decoy cells in urine (max: 90% of epithelial cells) were noted and a fourth biopsy at month seven showed BKPyVAN with positive multifocal SV40-positive nuclei, surprisingly together with moderate C4d in PTC (C4d2). Mild chronic interstitial fibrosis was now even more diffuse (ci3, affecting 100% of cortex), tubulitis and interstitial inflammation (i1, t3, ti1) were present, but in the absence of clinical deterioration were interpreted as signs of resolving BKPyVAN. Immunosuppression had already been reduced and BK-viremia together with decoy cell amount resolved gradually. Graft function stabilized at a creatinine of 1.6 mg/dL without any proteinuria. Since 2019 our patient was followed up at a remote center, where lung cancer was diagnosed and he died in mid-2020 with a functioning allograft. The serum creatinine and anti-Fy^a^ antibody titer trajectories in the context of biopsy results are provided in Figure 1. Detailed biopsy findings and representative histologic images are provided in the Supplementary Appendix, Supplementary Table S1 and Supplementary Figures S1A–D.

**FIGURE 2:**
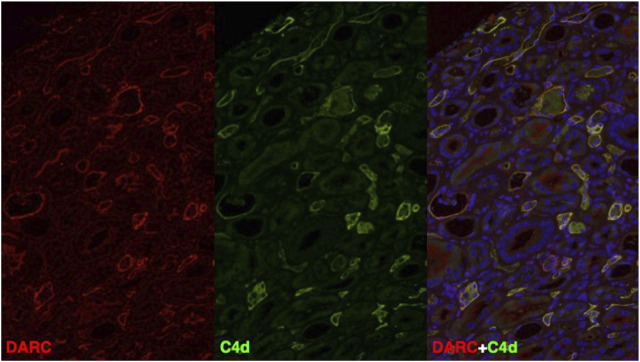
DARC (red) and C4d (green) and DAPI (blue) immunofluorescence staining in a representative biopsy specimen (Bx No. 3) fulfilling the Banff 2022 criteria for antibody-mediated rejection. On the right, double immunofluorescence is shown, where all areas with co-localization staining of DARC and C4d show high overlap (yellow = double positive) with respect to the positive area in PTC. Abbreviations: C4d, complement split product C4d; DARC, Duffy antigen-receptor of chemokines.

Due to the continuous presence of MVI in PTC without anti-HLA DSA but with endothelial C4d deposition, a causal relationship with the donor-specific anti-Fy^a^ antibodies was suspected and we therefore retrospectively performed double immunofluorescence for C4d and DARC in all four biopsies. In all three biopsies with linear C4d deposition, C4d was only positive in PTC, but not in glomeruli. DARC staining was only positive in PTC and absent in glomeruli. Most strikingly, and as illustrated in Figure 2 and Supplementary Figure S2, C4d and DARC showed a high degree of co-localization and similar staining intensity. This finding was considered highly suggestive of anti-DARC antibody-mediated C4d deposition in PTC.

**FIGURE 3:**
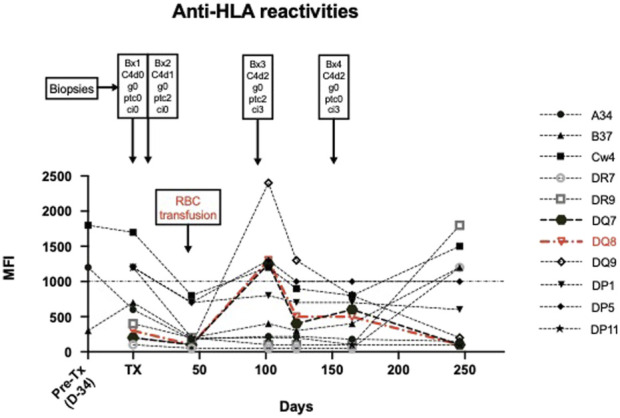
Illustrates anti-HLA reactivities over time in the context of biopsy results and the administration of RBC transfusions. Donor-specific antibody reactivities are shown in Red. A detailed discussion on the interpretation of SAB findings can be found in the Supplementary Appendix.

In order to corroborate our suspicion of ABMR mediated through anti-DARC antibodies, we performed detailed retrospective analysis of sera and NK cell genetics to rule out other contributors for MVI. As depicted in Figure 3 and shown in Supplementary Tables S2, S3 we were able to identify a transient and short-lived low-level anti-DQ8 DSA (MFI 1,300) around month three. Next, we retrospectively performed serial B and T FXCM using frozen cells from the kidney donor in order to pinpoint SAB results to biological relevance. We found that the transient DQ8 reactivity also coincided with a positive B cell FXCM (Supplementary Figures S3A–G), all other FXCM remained negative. Clinical workup showed that the occurrence of anti-DQ reactivities was closely related to the administration of RBC’s (Figure 3) and disappeared spontaneously without any treatment. A thorough description and interpretation of our SAB findings can be found in the Supplementary Appendix.

The option of MVI potentially being triggered by missing self, prompted us to perform donor/recipient KIR typing that revealed a high missing-self constellation for two established ligands (A11/KIR3DL2 and C2/KIR2DL1, Supplementary Table S4).

In follow-up biopsies MVI and C4d remained constantly high and interstitial fibrosis progressed without overt functional graft deterioration, again with no sign of glomerulitis or chronic glomerular lesions.

## Discussion

This is the first description of a potential *in vivo* relationship between the non-HLA antigen DARC, preformed anti-Duffy antibodies and histologic ABMR features in a kidney transplant. Immunofluorescent double-labelling revealing co-localization of DARC and C4d further supported anti-Duffy antibodies as potential cause of ABMR.

The combination of a recipient with preformed anti-Fy^a^ antibodies receiving an Fy^a^-positive donor kidney in the absence of anti-HLA alloreactivity is a rare event. We found only one report of a patient with preformed anti-Duffy antibodies who developed CDCXM-negative mixed rejection with crescentic GN, rendering the interpretation of Duffy-specific ABMR without SAB testing almost impossible as also upregulation of DARC during crescentic GN has already been demonstrated [20, 21].

Our findings are consistent with DARC acting as a non-HLA antigen potentially leading to clinically relevant rejection in kidney allografts. Our observation that interstitial fibrosis increased rapidly over time does confirm our previous findings showing that DARC expression on the transcriptome level, but also in IHC correlated well with fibrosis [11]. However, this study included patients with late ABMR, where DARC expression was investigated as a surrogate marker for ABMR and not as primary alloantibody target. DARC expression may lead to increases in cytokine levels within capillaries and thereby attract immune cells contributing to inflammation and potentially enhance subsequent fibrosis [22, 23]. One may argue that in this specific situation, preventive measures such as plasmapheresis or immunoadsorption could have been applied to prevent rejection due to anti-Fy^a^ antibodies, whereas declining this organ in order to wait for a Fy^a^-negative donor (approximately 32% of Caucasians) would not have been an ideal option [24]. With respect to the literature we found no strong recommendation regarding desensitization, but our report indicates that it may be warranted in such a case [15].

The co-localization of DARC and C4d within PTC suggests that DARC might be a relevant non-HLA antigen, with anti-Fy^a^ antibodies being capable of mediating ABMR. However, a caveat to our hypothesis is the fact that patients with anti-Fy^a^ antibodies, generally sensitized by blood transfusions, have a chance to also be sensitized against HLA. In our case in-depth analysis indeed revealed a low-level anti-DQ8 DSA after administration of RBC transfusions early posttransplant, which was confirmed by corresponding B cell FXCM reactivity. However, subsequently all anti-DQ reactivities faded without treatment, which is not supporting the occurrence of a memory response or *de novo* DSA formation. The appearance of transfusion-specific anti-HLA class II antibodies that also may act as DSA has been demonstrated earlier, but it has not been documented whether these reactions cause long-term or only short-term memory as it happened in our case [25]. In addition, this also did not lead to clinical deterioration, new onset of proteinuria or aggravation of MVI, even though we retrospectively demonstrated a high degree of “missing-self” constellation. It is interesting that despite having two degrees of missing-self, this failed to manifest glomerular MVI, making DARC expression the primary reason for MVI in PTC. A possible explanation for a milder course of ABMR due to anti-Duffy antibodies might be the fact, that DARC is - if at all - only very weakly expressed in glomeruli, which would explain the lack of proteinuria despite early renal deterioration in our patient [12]. This is also supported by the absence of glomerulitis, and C4d deposition being exclusively restricted to the PTC compartment.

This report illustrates the challenges associated with establishing the diagnosis of non-HLA-mediated ABMR on the basis of an actual clinical case. Despite having made use of our full current diagnostic armamentarium - with the exception of a lack of molecular biopsy diagnostics due to insufficient remaining material - and in order to perform a deep dive into the different potential immunologic processes that might have contributed to MVI in our patient, it seems almost impossible to provide definite evidence for a causal relationship between a non-HLA DSA and MVI in our representative case. Of note, the different potential triggers of MVI are not mutually exclusive, and other conditioning factors such as DGF and ischemia might also have played a role in this specific scenario.

Nevertheless, our findings strengthen a potential association of preformed anti-Fy^a^ antibodies with MVI, but our report also highlights the caution that is warranted to exclude the remaining causes of MVI, rendering it a highly complex task to prove causality [26].

## Data Availability

Original datasets are available upon request to the corresponding author.
