# Supplementary material for: DARC and Anti-Duffy Antibodies in the Line of Fire: The Challenges in Pinpointing the Etiology of Microcirculation Inflammation to a Distinct Entity
Source: Transpl Int. 2026 Jan 12;38:15601. doi: 10.3389/ti.2025.15601 (PMC12832551; doi:10.3389/ti.2025.15601)

**Supplementary appendix:**

**DARC and anti-Duffy antibodies in the line of fire: The challenges in pinpointing the etiology of microcirculation inflammation to a distinct entity.**

Farsad Eskandary^1^*, Günther F. Körmöczi^2^, Marlies Schönbacher^2^, Gottfried Fischer^2^, Ingrid Faé^2^, Sabine Wenda^2^, Daniela Koren^2^, Rainer Oberbauer^1^, Roman Reindl-Schwaighofer^1^, Andreas Heinzel^1^, Johannes Kläger^3^, Nicolas Kozakowski^3^, Stephan Segerer^4^, Konstantin Doberer^1^, Luis G. Hidalgo^5^, Helga Schachner^3^, Georg A. Böhmig^1^ and Heinz Regele^3^

^1^ Medical University of Vienna, Department of Medicine III, Division of Nephrology and Dialysis, Vienna, Austria

^2^ Medical University of Vienna, Department of Blood Group Serology and Transfusion Medicine, Vienna, Austria

^3^ Medical University of Vienna, Department of Pathology, Vienna, Austria

^4^Division of Nephrology, Dialysis and Transplantation, Kantonsspital Aarau, Switzerland

^5^Histocompatibility and Immunogenetics Laboratory, University of Alabama at Birmingham, Alabama, USA

**^*^**Correspondence:

Farsad Eskandary, Department of Nephrology and Dialysis, Medical University of Vienna, Währinger Gürtel 18-20, 1090 Vienna, Austria, [farsad.eskandary@meduniwien.ac.at](mailto:farsad.eskandary@meduniwien.ac.at)

Supplementary methods:

Double immunofluorescence of DARC and C4d:

Double immunofluorescence was performed simultaneously on paraffin-embedded sections. After deparaffinization and heat-pretreatment in citrate-buffer at pH 6 in an autoclave at 1bar for 20min, mouse anti-DARC (dilution 1:100) and rabbit anti-C4d (dilution 1:2000) were incubated at +4°C overnight. After washing in PBS 3 times, incubation with and goat anti-mouse Alexa 594 (A11005-Molecular Probes Inc.) and goat anti-rabbit ALEXA 488 (A11006 Molecular Probes Inc.) were added in dilution 1:500 for 60 min at room temperature. After washing in PBS 3 times a counterstain with DAPI (Serva: Cat.no. 18860.01) at a dilution 1:50 000 for 2 min and coverslip with Geltol (Thermo scientific Cat.no. 484950) was performed.

Anti-HLA antibody testing and high-resolution HLA typing:

HLA antibody detection was carried out using LABscreen single-antigen flow-bead assays (One Lambda, Canoga Park, CA). All serum samples were incubated with 10mM ethylenediaminetetraacetic acid (EDTA) to account for complement interference. For data acquisition we used a LABScan^TM^ 1000 flow analyzer (Luminex Corporation, Austin, TX, USA). All test results were recorded as mean fluorescence intensity (MFI) with an MFI threshold >1,000 considered as positive. NC bead and autoreactive bead MFI were subtracted from all results to avoid false-positive findings. Donor-specificity was defined according high-resolution donor/recipient HLA typing (HLA-A, -B, -Cw, -DR, -DQ, -DP), which was also done in our HLA laboratory using next generation sequencing (NGS). For NGS analyses, long-range amplification of the whole HLA genes was achieved using in-house primers. Amplification was performed using GoTaq Long polymerase chain reaction (PCR) Mastermix (Promega Corporation, Woods Hollow Roads Madison). Library preparation was performed according to the manufacturer’s instructions. Size selection was achieved on an E-Gel (Invitrogen, Kiryat Shoma, Israel), only fragments with sizes >400 bp were selected. After quantification, 26 PMol of fragments were used for emulsion PCR, enriched Ion sphere particles were loaded onto an Ion Torrent 520 chip in an Ion Chief device (LifeTechnologies, Carlsbad, California) and subsequently sequenced on an S5 device (LifeTechnologies, Carlsbad, California) with a flow number of 850, for 400 base reads. Analysis of the reads was performed using two different NGS analysis software packets (TypeStream NGS Analysis Software, One Lambda, Inc. Canoga Park CA; NGSengine, GenDX, Utrecht, The Netherlands).

KIR Typing:

The repertoires of the inhibitory KIR receptors including 2DL1, 2DL2, 2DL3, 2DL4, 2DL5, 3DL1, 3DL2 and the activating receptors 2DS1, 2DS2, 2DS3, 2DS4, 2DS5, 3DL3, 3DS1, 2DP1, 3DP1 were genotyped using the Olerup SSP KIRGenotyping Kit (CareDx Inc). We amplified highly pure DNA through PCR using sequence-specific primers, following the manufacturer's instructions. Electrophoresis was conducted using 2% E-Gel Agarose Gels with SYBR-Safe DNA GelStain. Gels were visualized by an Electrophoresis Device (Perfect Blue Maxi, Peqlab Biotechnoloy). KIR typing results were interpreted by employing validated probes obtained from the international KIR Exchange program (Immunogenetics, Center ofthe University of California at Los Angeles).

FXCM:

*Sera preparation:*

All sera were treated with EDTA avoiding prozone effect and then frozen at -80°C. Upon use sera were thawed and centrifuged at 14000g for 10 minutes.

*Cell preparation:*

Mononuclear cells from spleen were isolated with LymphoprepTM density medium and frozen in liquid nitrogen. After thawing, 1x10^7^ lymphocytes were suspended in 200μL PBS containing 1M MgCl and were incubated with 500μL Pronase-working solution (Sigma-Aldrich, Saint Louis, USA, Ref P8811-100mg) for 30 minutes at 37°C (Pronase end-concentration: 1.0mg/mL). After digestion with pronase, the cells were incubated for at least 1 minute with DNAse (Deoxyribonuclease I Sigma Aldrich, Saint Louis, USA, Ref D5025-15K) at a concentration of 500kUnits. Subsequently, cells were washed three times with PBS containing 10% FCS and resuspended in PBS containing 2% FCS and 0.1% NaN_3_ and 1mM EDTA.

*FXCM procedure:*

15μL serum (patient and control sera) was pipetted in a WhatmanTM Microplate (GE Healthcare UK Limited, Buckinghamshire, UK, Ref 7701-3250) and 15μL lymphocyte-suspension were added. Negative and positive control sera were added appropriately, all test samples were done in duplicate and an additional background control was added by incubating undigested lymphocytes with negative control serum. After vortexing and incubation for 30 minutes in the dark at 4°C, several washing steps including PBS with 2%FCS, 0.1% NaN_3_ and 1mM EDTA were performed.

After removal of all buffers and drying, 50μL monoclonal antibody mix (Dilution: 1:512) was added to the wells including CD3 eFluor® 450 (Clone SK7, eBioscience Inc, San Diego, CA, USA, Ref 9048-0036-120), CD19 APC (Clone SJ25C1, eBioscience Inc, San Diego, CA, USA, Ref 9017-0198-120) and AffiniPure F(ab´)2 Fragment Goat Anti-Human IgG, Fcg Fragment Specific PE (Jackson ImmunoResearch, West Grove, PA, Ref 109-116-098). All antibodies were incubated at once for 10 minutes in the dark at room temperature. After further washing, lymphocytes were resuspended in 120μL PBS containing 2% FCS and 0.1% NaN_3_ and 1mM EDTA and were additionally spiked for 10 minutes with 7-AAD (BD PharmingenTM, Ref 559925) to exclude dead cells, which was followed by immediate acquisition on the flow-cytometer. Controls were checked for enzyme digestion and to ensure comparable HLA expressivity on all samples. For these purposes antibody mixes also containing CD16 (low-affinity receptor for IgG PE eBioscience Inc, San Diego, CA, USA, Ref 9012-0168-025), Anti-Human HLA-ABC FITC (Clone W6/32, eBioscience Inc, San Diego, CA, USA, Ref 11-9983) and Anti-Human HLA-DR PE (Clone L243, eBioscience Inc, San Diego, CA, USA, Ref 12-9952) were used, all other steps were equal to the above-described test protocol.

*FXCM readout:* Flow cytometric analysis of anti-human IgG-fluorescence was conducted by linear data acquisition where no linearization via channel-shift transformation is required. By adjusting the voltage amplification of the measurement parameter, weakly positive signals are effectively detected. The data are directly evaluated against the positive threshold, defined as 6000 above the MFI of the negative control.

Supplementary results:

Suppl. Table S1: Detailed biopsy results graded according to Banff 2022 update:

| **Biopsies** | **g** | **ptc** | **C4d** | **i** | **ti** | **t** | **ah** | **mm** | **cg** | **ci** | **cv** | **ct** | **v** | **SV40** | **MLPTC** | **Banff 2022 diagnosis** |
| --- | --- | --- | --- | --- | --- | --- | --- | --- | --- | --- | --- | --- | --- | --- | --- | --- |
| Bx1 (d3)***** | **0** | **0** | **0** | 0 | 0 | 0 | x | 0 | 0 | 0 | x | 0 | x | n.d. | n.d. | No AMR |
| Bx2 (d14) | **0** | **2** | **1** | 0 | 0 | 0 | 0 | 0 | 0 | 0 | 0 | 0 | 0 | n.d. | n.d. | Active AMR |
| Bx3 (d102) | **0** | **2** | **2** | 0 | 0 | 0 | 1 | 0 | 0 | 3 | 2 | 0 | 0 | n.d. | 0 | Active AMR |
| Bx4 (d163) | **0** | **n.a.**** | **2** | 1 | 1 | 3 | 0 | 0 | 0 | 3 | 0 | 0 | 0 | pos | n.d. | No AMR  (C4d+ and BKPyVAN) |

*Biopsy specimen containing only marginal cortex material; **n.a., not applicable, since positive SV40 and BKPyVAN with resolving interstitial infiltrate omitted ptc assessment.

Abbreviations: AMR, antibody-mediated rejection; n.d., not done; MLPTC, multilayering of peritubular capillaries in electron microscopy; SV40, simian virus 40 stain in immunohistochemistry.

Suppl. Table S2: HLA-typing results, HLA mismatches are shown in Yellow.

| **HLA class I** | **A** | **A** | **B** | **B** | **Cw** | **Cw** |
| --- | --- | --- | --- | --- | --- | --- |
| **Donor** | 02:01 | 02:01 | 07:02 (Bw6) | 08:01 (Bw6) | 07:01 | 07:02 |
| **Recipient** | 02:01/09 | 11:01 | 07:02 (Bw6) | 40:02 (Bw6) | 02:02 | 07:02 |

| **HLA class II** | **DRB1** | **DRB1** | **DRB4** | **DRB5** |
| --- | --- | --- | --- | --- |
| **Donor** | 15:01 | 04:04 | 01:03 | 01:01 |
| **Recipient** | 15:01 | 15:02 |  | 01:01; 01:02/08 |

| **HLA class II** | **DQA1** | **DQA1** | **DQB1** | **DQB1** | **DP** | **DP** |
| --- | --- | --- | --- | --- | --- | --- |
| **Donor** | 01:02 | 03:01 | 03:02 | 06:02 | 02:01 | 04:01 |
| **Recipient** | 01:02 | 01:03 | 06:01 | 06:02 | 02:01 | 05:01 |

Abbreviations: HLA, human leukocyte antigen

Eplet mismatch was calculated using HLA Fusion Software version 4.2:

HLA class I (A, B & Cw) eplet mismatch: 9

HLA class II (DR, DQ & DP) eplet mismatch: 35

Suppl. Table S3: Luminex results are given for any anti-HLA reactivity that showed a single MFI >1,000 (Yellow) over the course of recordings. Any reactivity with potential donor-specificity with a threshold of MFI >1,000 is shown in Red.

| **Luminex Class I:** | **04.04.17** | **08.05.17 (TX)** | **21.06.17** | **18.08.17** | **08.09.17** | **20.10.17** | **09.01.18** |
| --- | --- | --- | --- | --- | --- | --- | --- |
| Locus | A34 | A34 | A34 | A34 | A34 | A34 | A34 |
| MFI | 1200 | 600 | 180 | 220 | 220 | 180 | 150 |
| Pot. DSA? | No | No | No | No | No | No | No |
| Locus | Cw4 | Cw4 | Cw4 | Cw4 | Cw4 | Cw4 | Cw4 |
| MFI | 1800 | 1700 | 800 | 1200 | 900 | 800 | 1500 |
| Pot. DSA? | No | No | No | No | No | No | No |
| Locus | B37 | B37 | B37 | B37 | B37 | B37 | B37 |
| MFI | 300 | 700 | 200 | 400 | 300 | 400 | 1200 |
| Pot. DSA? | No | No | No | No | No | No | No |
| NC bead | 94 | 82 | 22 | 44 | 27 | 25 | 63 |
| **Luminex Class II:** | **04.04.17** | **08.05.17 (TX)** | **21.06.17** | **18.08.17** | **08.09.17** | **20.10.17** | **09.01.18** |
| Locus | DP1 | DP1 | DP1 | DP1 | DP1 | DP1 | DP1 |
| MFI | n.d. | 1200 | 700 | 800 | 700 | 700 | 600 |
| Pot. DSA? | No | No | No | No | No | No | No |
| Locus | DP5 | DP5 | DP5 | DP5 | DP5 | DP5 | DP5 |
| MFI | n.d. | 1200 | 700 | 1300 | 1000 | 1000 | 1000 |
| Pot. DSA? | Auto | Auto | Auto | Auto | Auto | Auto | Auto |
| Locus | DP11 | DP11 | DP11 | DP11 | DP11 | DP11 | DP11 |
| MFI | n.d. | 1200 | 200 | 200 | 200 | 100 | 100 |
| Pot. DSA? | No | No | No | No | No | No | No |
| Locus | DQ7 | DQ7 | DQ7 | DQ7 | DQ7 | DQ7 | DQ7 |
| MFI | n.d. | 200 | 100 | 1200 | 400 | 600 | 100 |
| Pot. DSA? | No | No | No | No | No | No | No |
| Locus | DQ8 | DQ8 | DQ8 | **DQ8** | DQ8 | DQ8 | DQ8 |
| MFI | n.d. | 300 | 100 | **1300** | 500 | 500 | 100 |
| Pot. DSA? | Yes | Yes | Yes | **Yes** | Yes | Yes | Yes |
| Locus | DQ9 | DQ9 | DQ9 | DQ9 | DQ9 | DQ9 | DQ9 |
| MFI | n.d. | 200 | 100 | 2400 | 1300 | 800 | 200 |
| Pot. DSA? | No | No | No | No | No | No | No |
| Locus | DR7 | DR7 | DR7 | DR7 | DR7 | DR7 | DR7 |
| MFI | n.d. | 100 | 50 | 50 | 50 | 50 | 1200 |
| Pot. DSA? | No | No | No | No | No | No | No |
| Locus | DR9 | DR9 | DR9 | DR9 | DR9 | DR9 | DR9 |
| MFI | n.d. | 400 | 200 | 100 | 100 | 100 | 1800 |
| Pot. DSA? | No | No | No | No | No | No | No |
| NC bead | n.d. | 64 | 18 | 45 | 32 | 31 | 70 |
| **MICA screen:** | n.d. | **negative** | n.d. | n.d. | n.d. | n.d. | n.d. |
| **Luminex Screen:** |  |  |  |  |  |  |  |
| **Class I** | pos. | pos. | n.d. | n.d. | n.d. | n.d. | n.d. |
| **Class II** | neg. | pos. | n.d. | n.d. | n.d. | n.d. | n.d. |

Abbreviations: DSA, donor-specific antibody; MFI, mean fluorescence intensity; MICA, MHC class I chain-related gene A; NC, negative control; TX, transplantation

Suppl. Table S4: HLA class I typing results and KIR typing results with respect to missing-self are shown in Red.

| **HLA class I typing** | **A** | **A** | **B** | **B** | **Cw** | **Cw** |
| --- | --- | --- | --- | --- | --- | --- |
| **Donor** | 02:01 | 02:01 | 07:02 (Bw6) | 08:01 (Bw6) | 07:01 (C1) | 07:02 (C1) |
| **Recipient** | 02:01/09 | 11:01 | 07:02 (Bw6) | 40:02 (Bw6) | 02:02 (C2) | 07:02 (C1) |
| **KIR typing** | KIR3DL2 | KIR3DL2 | KIR3DL1 | KIR3DL1 | KIR2DL1 | KIR2DL1 |
| **Donor KIR** | pos | pos | pos | pos | pos | pos |
| **Recipient KIR** | pos | pos | pos | pos | pos | pos |
| **KIR ligand donor** | - | Missing (A3/11) | - | - | Missing (C2) | - |
| **KIR ligand recipient** | - | 3DL2/A11 | - | - | 2DL1/C2 | - |
| **Missing self** | NO | YES | NO | NO | YES | NO |
| **KIR typing** | - | - | - | - | KIR2DL2 | KIR2DL2 |
| **Donor KIR** | - | - | - | - | neg | neg |
| **Recipient KIR** | - | - | - | - | pos | pos |
| **KIR ligand donor** | - | - | - | - | 2DL2/C1 | 2DL2/C1 |
| **KIR ligand recipient** | - | - | - | - | 2DL2/C1 | 2DL2/C1 |
| **Missing self** | - | - | - | - | NO | NO |
| **KIR typing** | - | - | - | - | KIR2DL3 | KIR2DL3 |
| **Donor KIR** | - | - | - | - | pos | pos |
| **Recipient KIR** | - | - | - | - | pos | pos |
| **KIR ligand donor** | - | - | - | - | 2DL3/C1 | 2DL3/C1 |
| **KIR ligand recipient** | - | - | - | - | 2DL3/C1 | 2DL3/C1 |
| **Missing self** | - | - | - | - | NO | NO |

Abbreviations: HLA, human leukocyte antigen; KIR, killer cell immunoglobulin-like receptor

Interpretation of SAB results:

HLA antibodies were measured at seven different timepoints (**see supplementary table S3 for SAB details**). There was one single SAB measurement at the time of Bx3 (C4d2, ptc2, **see supplementary table S1** for more biopsy details), in which a reactivity above our center-specific MFI threshold >1000 against a single donor-specific bead (DQ8, Bead No.: 61, DQA1*02:01, DQB1*03:02) was recorded with an MFI of 1,300 (anti-DQ8). Overall, the reactivity pattern of this short lasting and self-limiting anti-HLA antibody flare encompassed all DQ3-associated beads, making an antibody against epitopes on the beta chain of DQ3 likely [Solitary Antibody against alpha chains of DQ unlikely, since all beads outside DQ3 with shared alpha chains were negative. However, DQA1*03:02 is present on beads 63 (DQ8) as well as 66 (DQ9) and had the highest MFI of all DQ3 beads making an additional DQA1 antibody or a shared epitope possible]. However, since at this measurement also an MFI of >1,000 occurred against a well-known often false-positive bead that was also self (DP5, Bead No. 70, DPA1*02:02, DPB1*05:01), the DQ result was not counted as positive and prompted us to observe the findings in further measurements regarding a potential anti-DQ8 DSA. At both earlier and later timepoints, no reactivity at all was recorded for bead no. 61 (DQ8), making it very unlikely to be of substantial significance. Analyzing this positive measurement further we found, that our patient had received two radiation-treated RBC transfusions on June 24, 2017, three days after the last SAB measurement, that was completely negative for any donor-specific anti-HLA antibody. It is very likely that these had induced a mild transient (donor-specific) anti-HLA reactivity without a memory response due to the short half-life of RBC’s and the accompanied un-sustained stimulation by HLA allo-antigens. Overall, the observed anti-DQ response completely vanished after a few months as depicted in **Figure 3 and presented in supplementary table S3**, making it very untypical for a true *de novo* anti-DQ3 DSA. It remains speculative, but the transient reactivity seemed to be more likely induced by exposure to DQ9 (not donor-specific, but maybe triggered by RBC transfusion), as the bead with the highest MFI was bead 66 (DQ9, MFI 2,400) and about four months after all anti-DQ reactivities had disappeared we recorded two non-donor-specific antibodies against DR7 and DR9, which are both in linkage disequilibrium with DQ9, making an association possible and DQ8 as a primary target less likely **(Figure 3 and supplementary table S3)**.

Suppl. Figure 1A, representative biopsy images.

Bx1: PAS
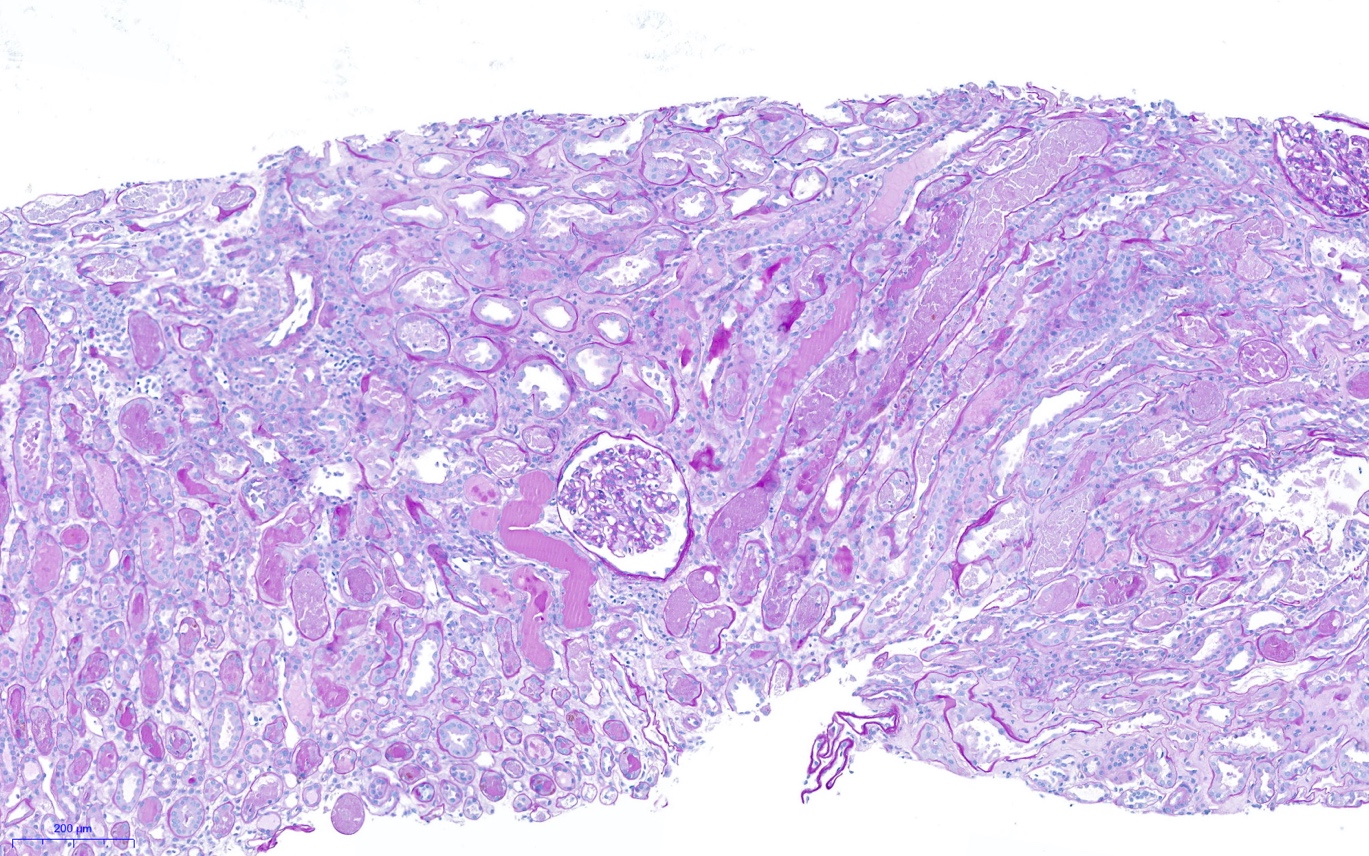


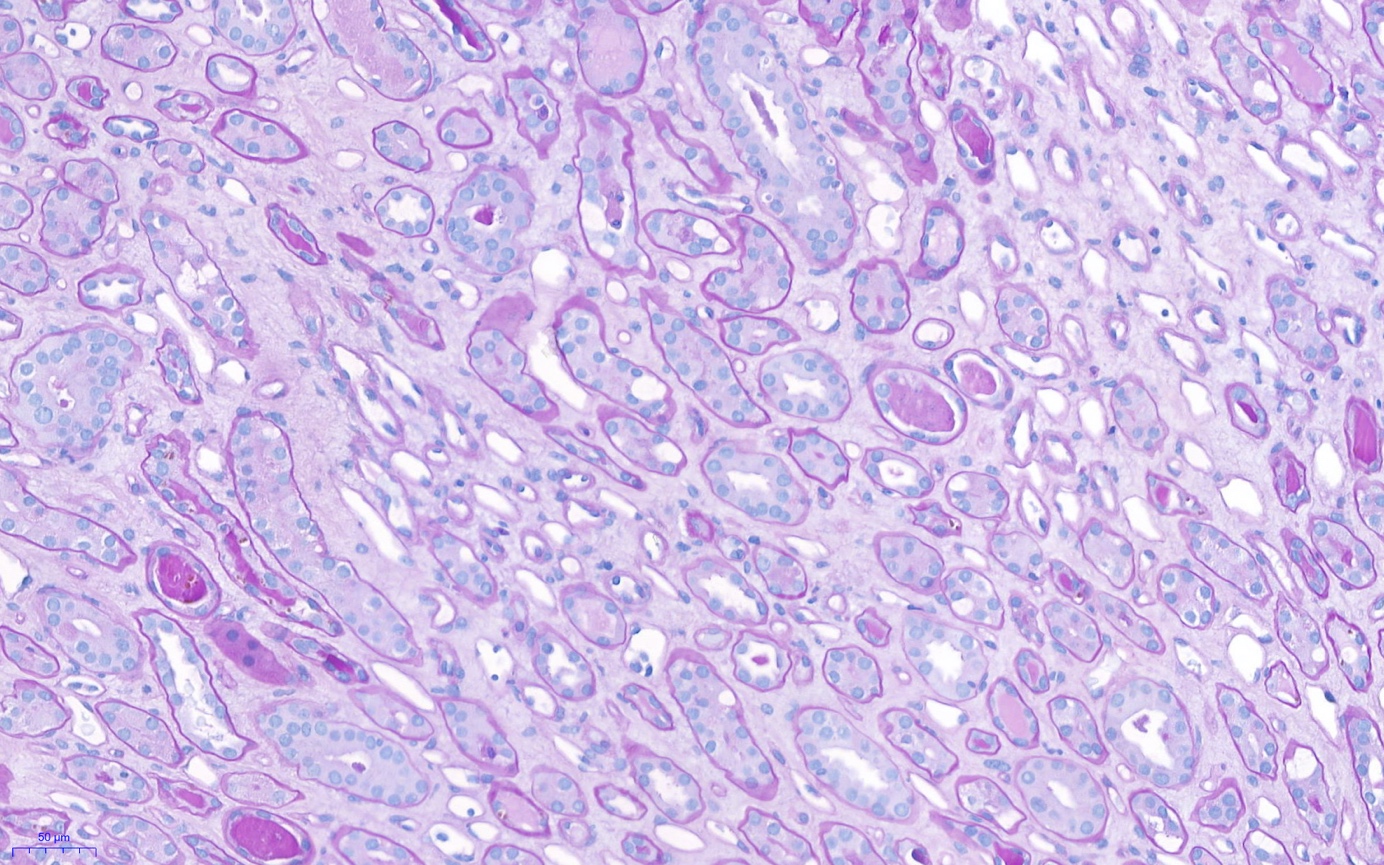


Suppl. Figure 1B, representative biopsy images.

Bx2: PAS and C4d


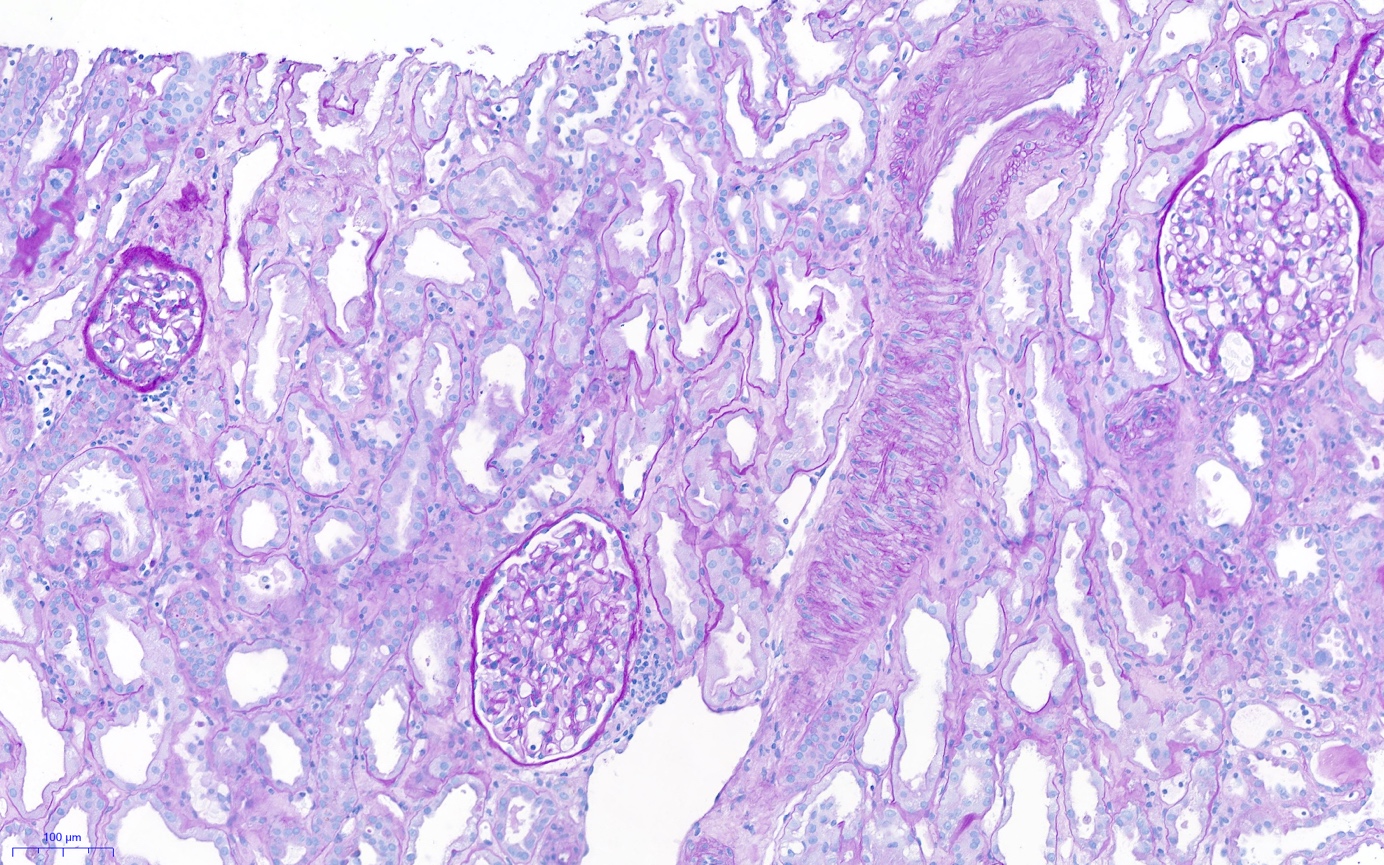


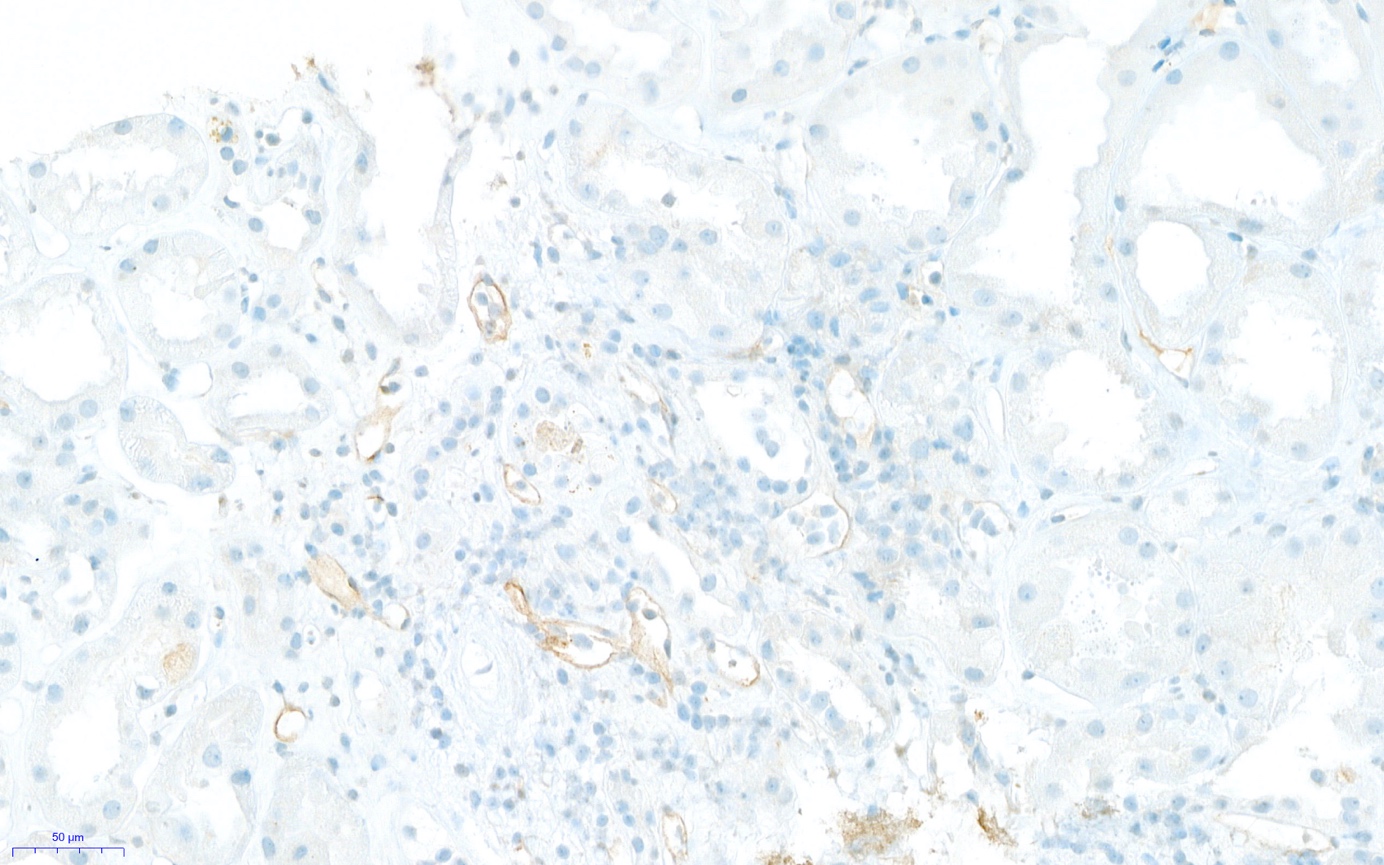


Suppl. Figure 1C, representative biopsy images.

Bx3: PAS


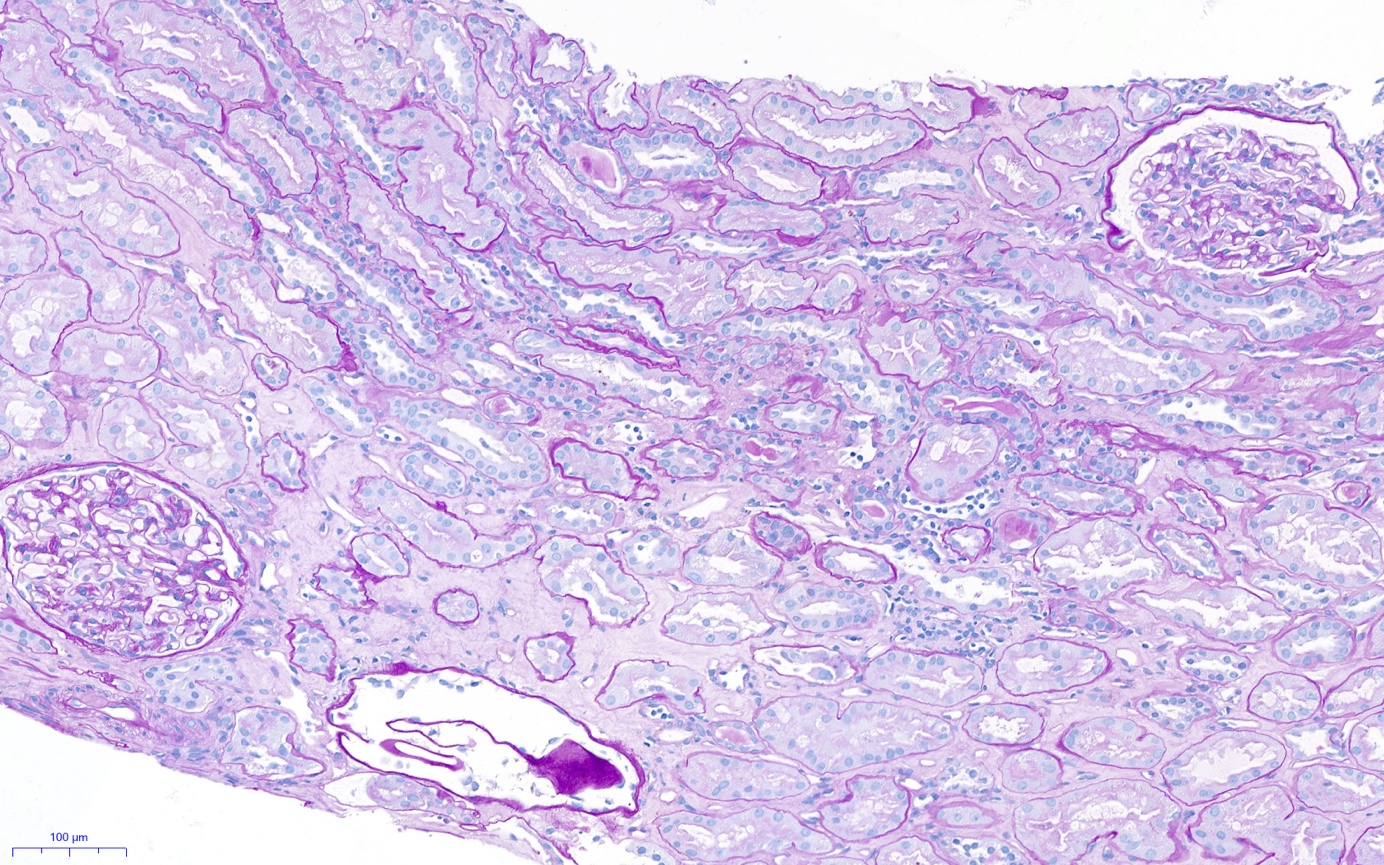


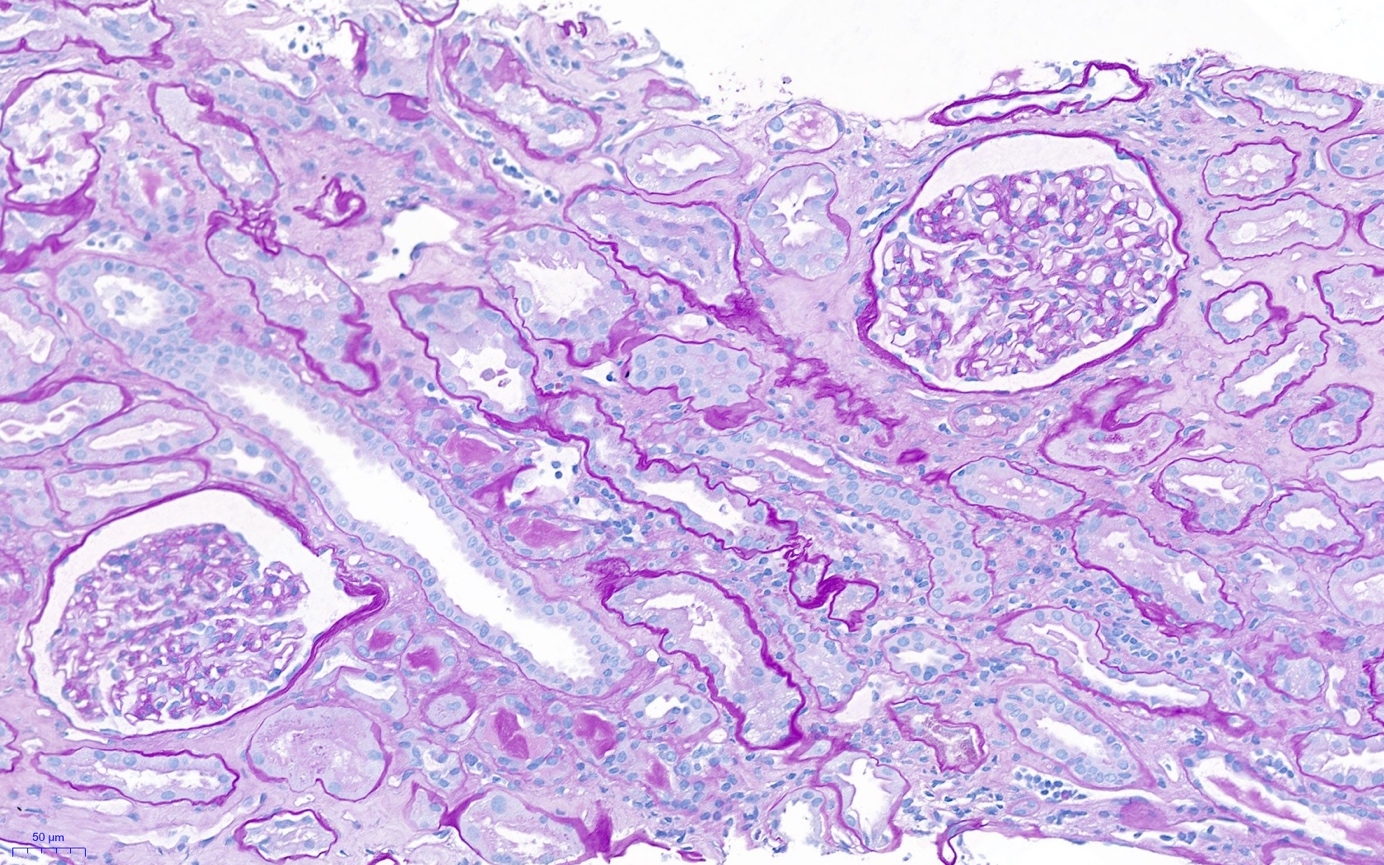


Suppl. Figure 1D, representative biopsy images.

Bx4: PAS, C4d and SV40


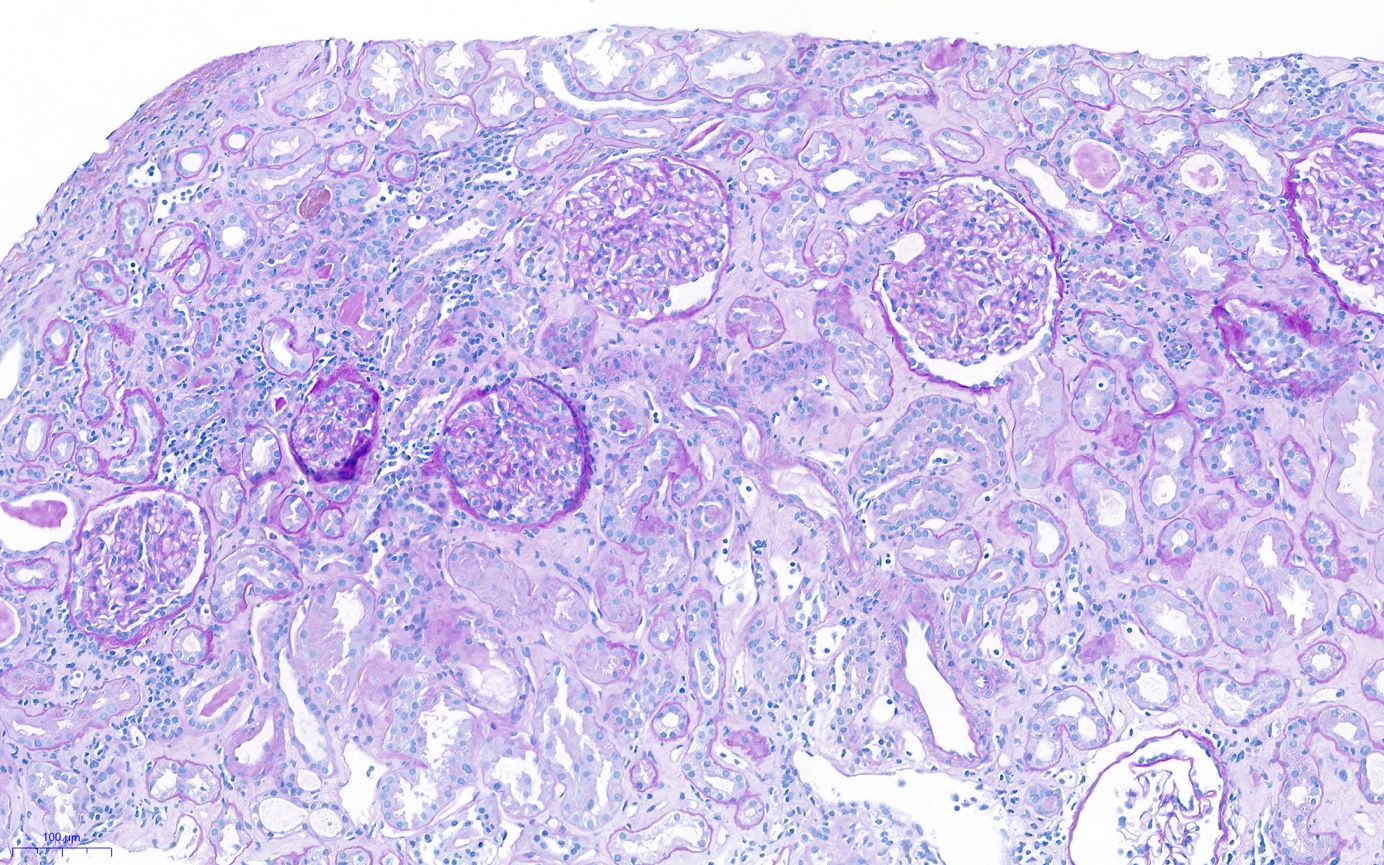


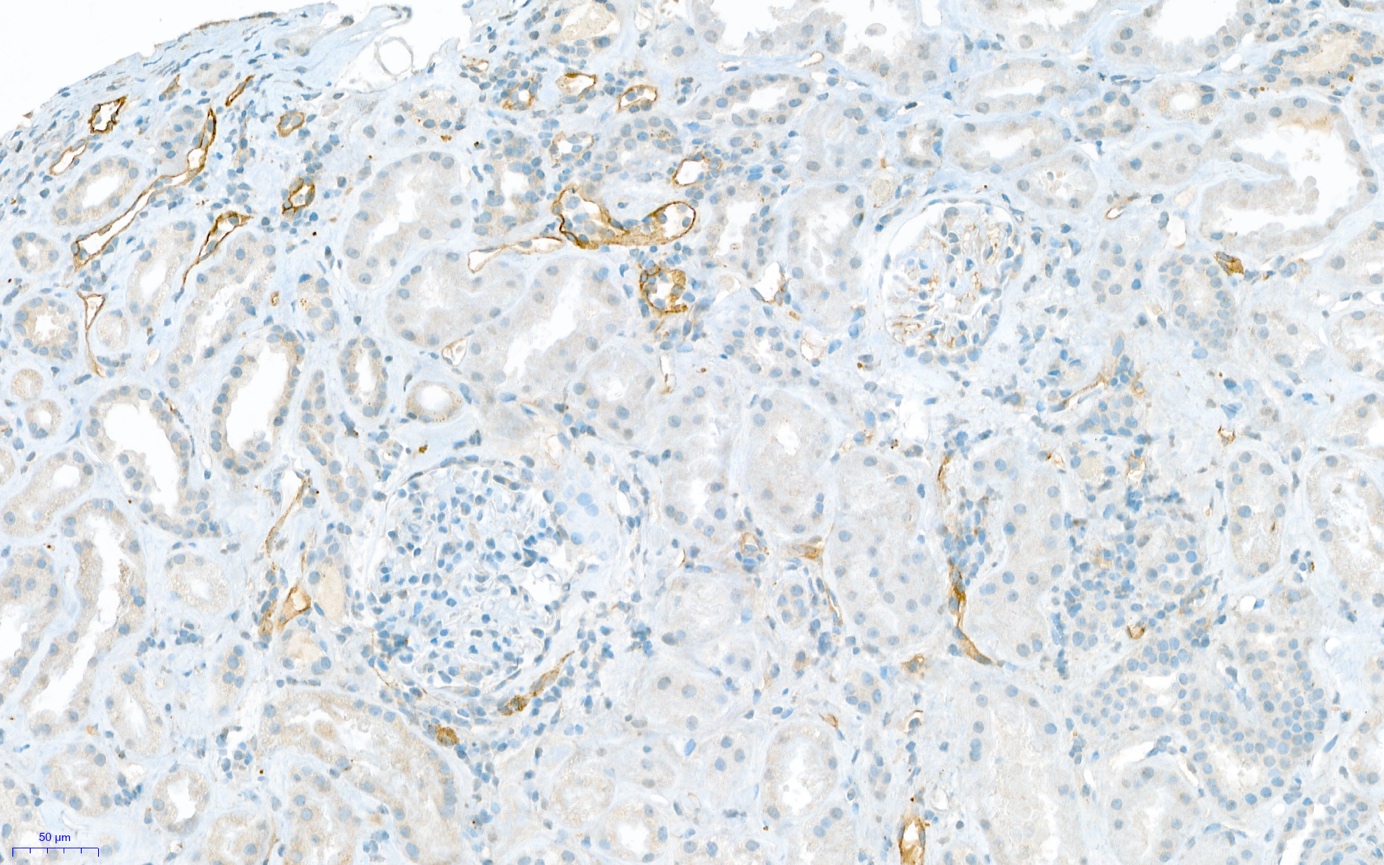


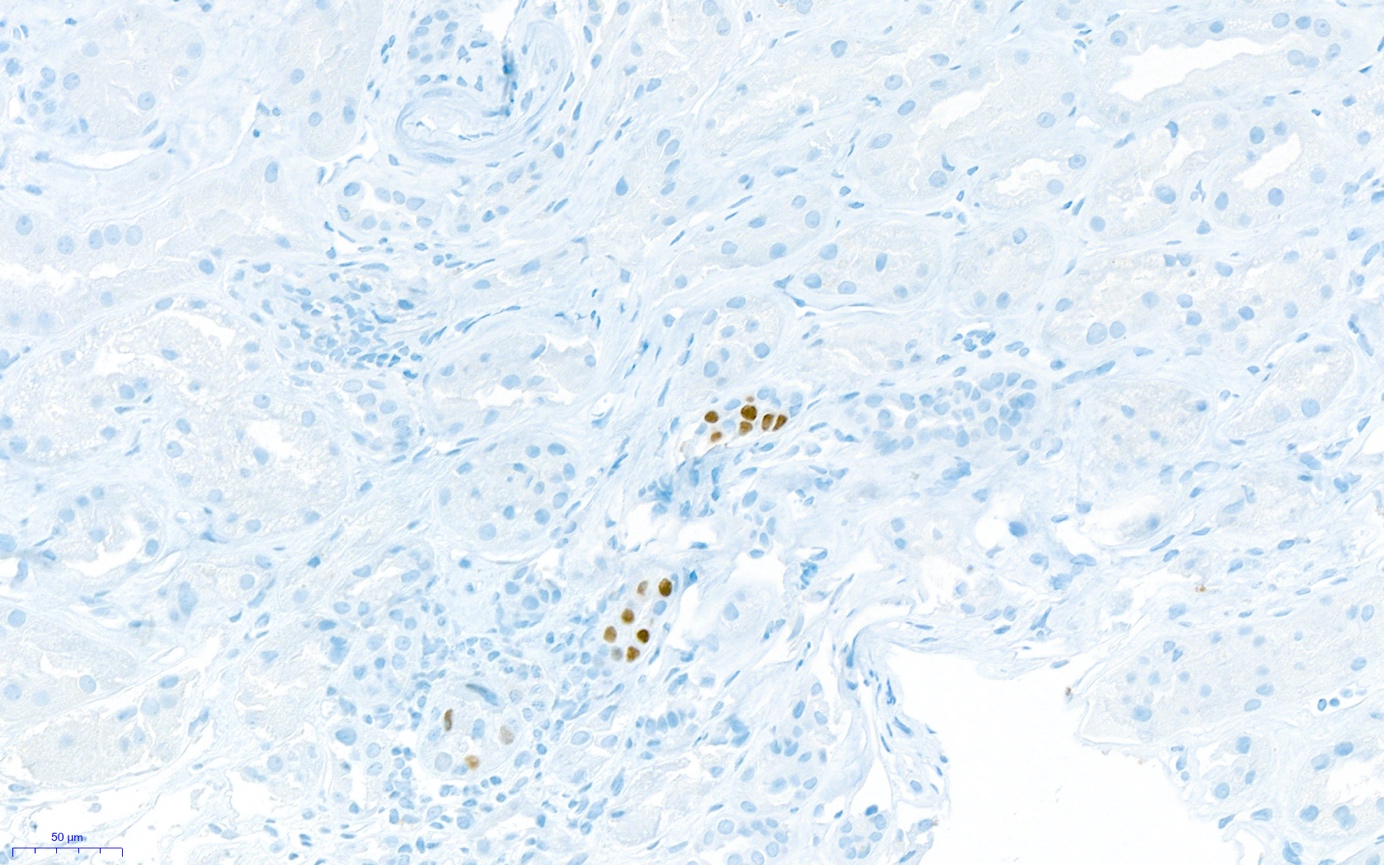


Suppl. Figure S2, representative biopsy images.

Bx4: DAPI (blue), C4d (green), DARC (red) & double IF for C4d/DARC (yellow, if double-positive) are shown. Note, that there is no detectable linear C4d staining in glomeruli (thick red arrows) when compared to PTC (thin red arrows)


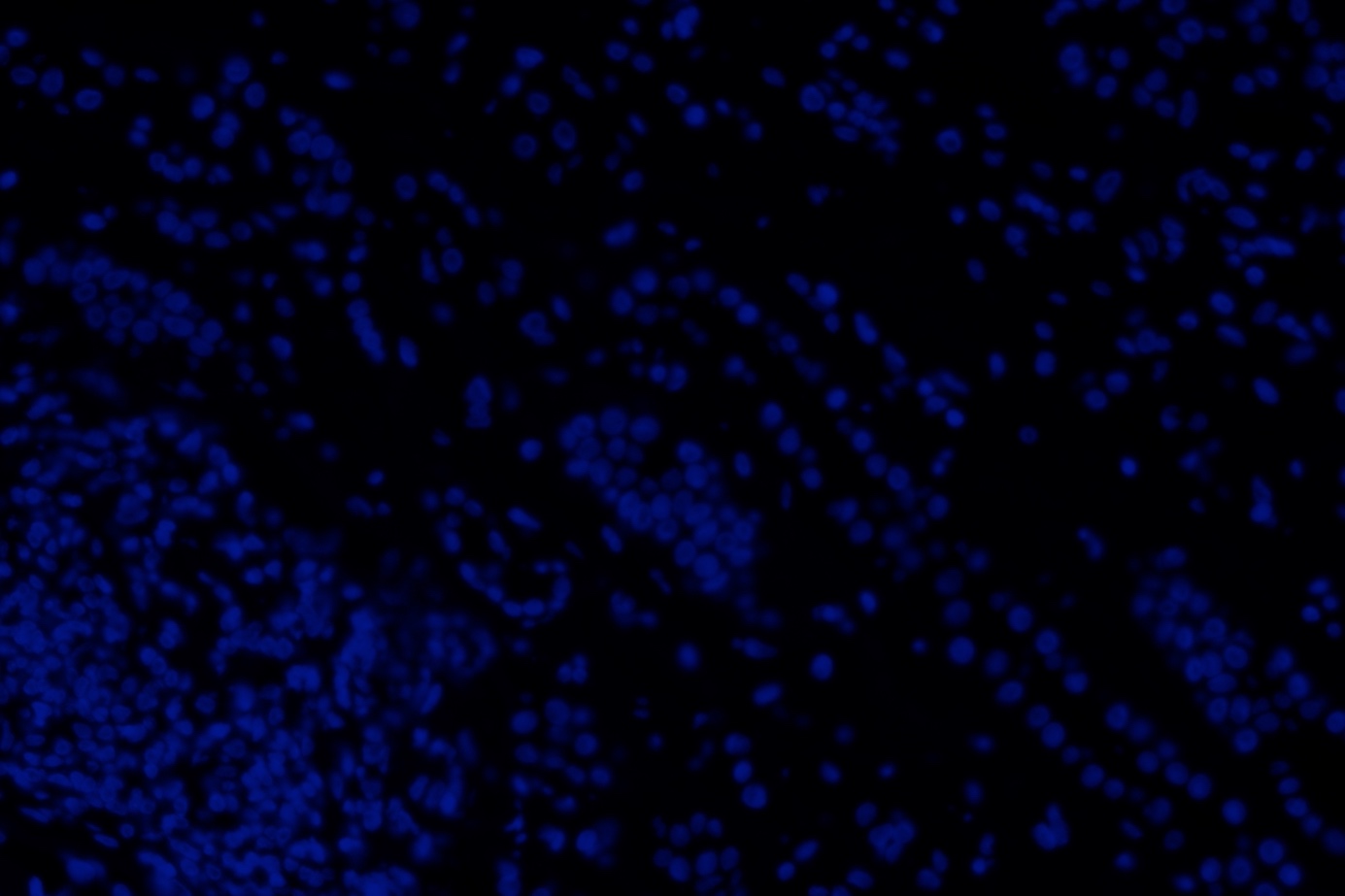


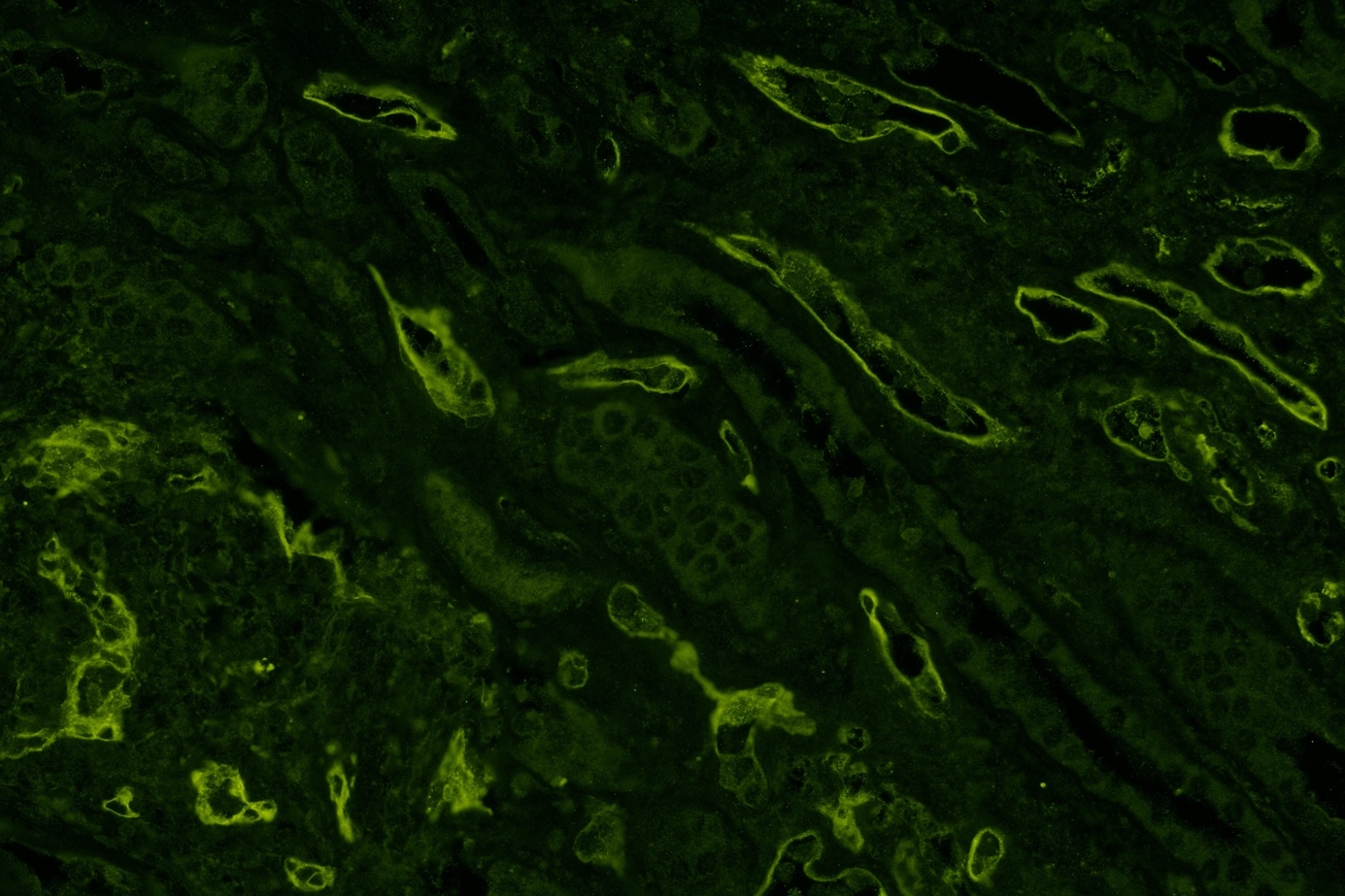


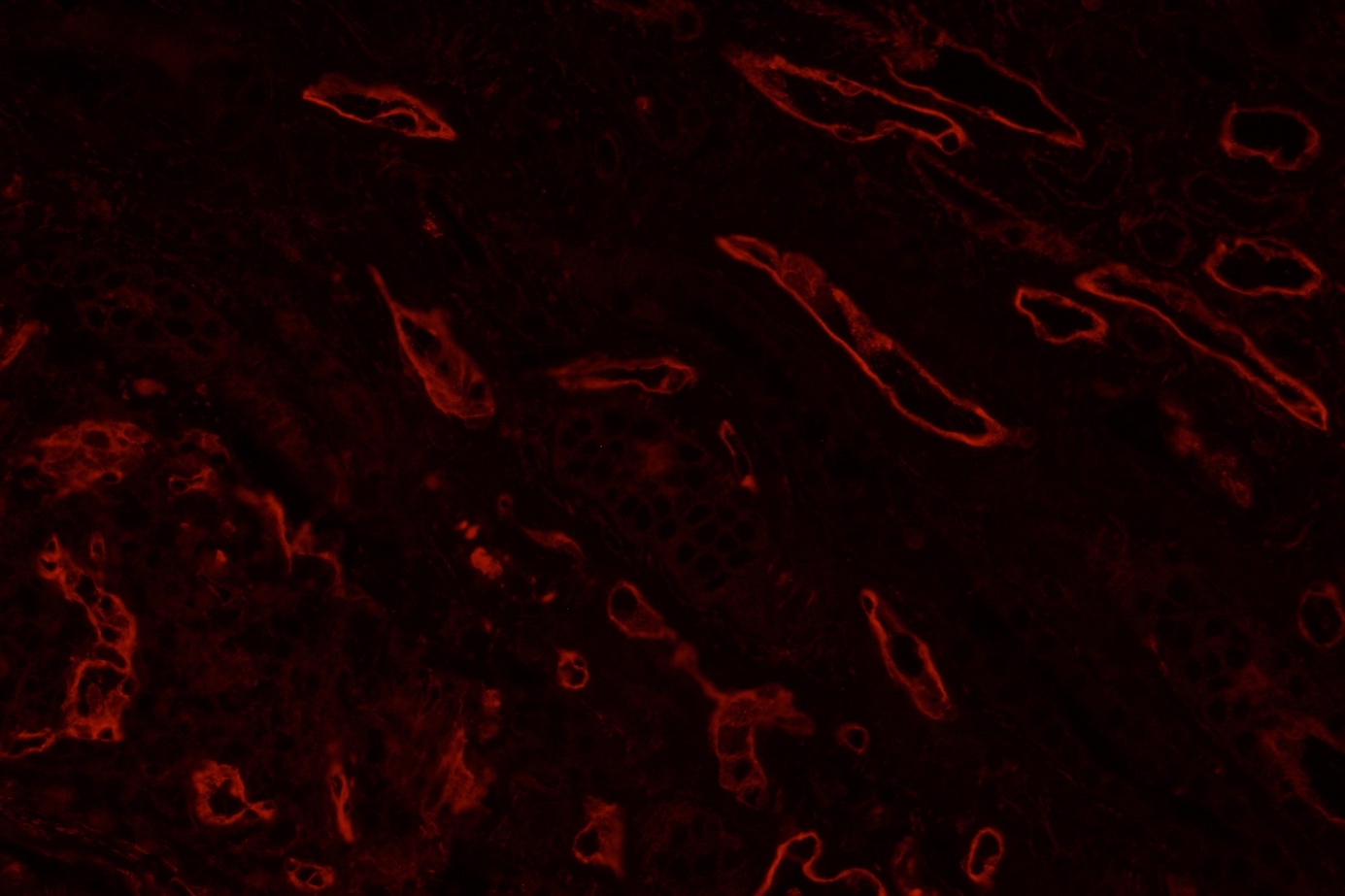


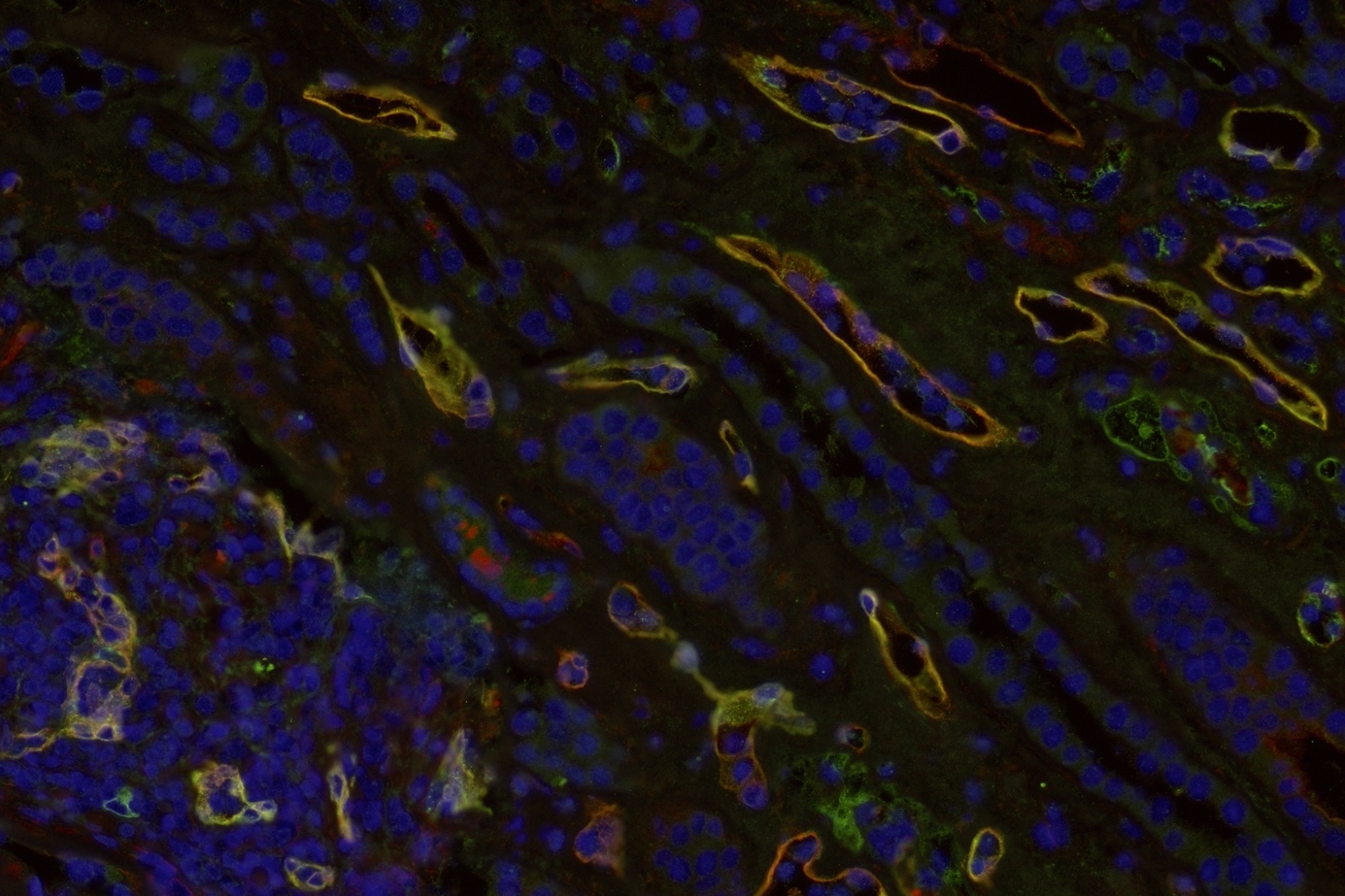


Supplementary Figure 3A-G: T and B cell FXCM results. Pictures on the left show T cell and pictures on the right show B cell FXCM results. Note the linear scale on the x-axis (x1000).

3A: negative control for T (left, green) and B (right, red) cell FXCM:


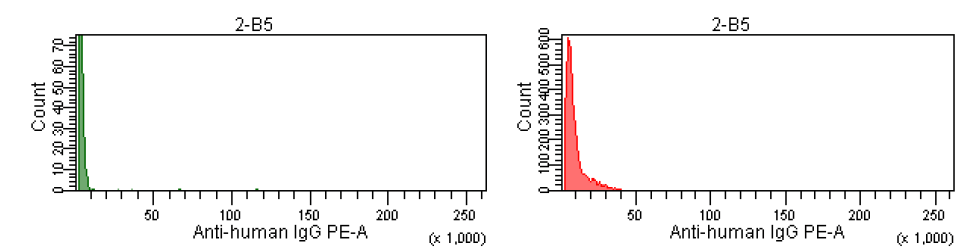


3B: positive control for T (left, green) and B (right, red) cell FXCM: note that the peak in the B cell FXCM is extremely skewed to the right translating to a MFI >250000. The pictogram below shows a B cell FXCM with the comparison between negative control (green, left side) and positive control (red, right side).


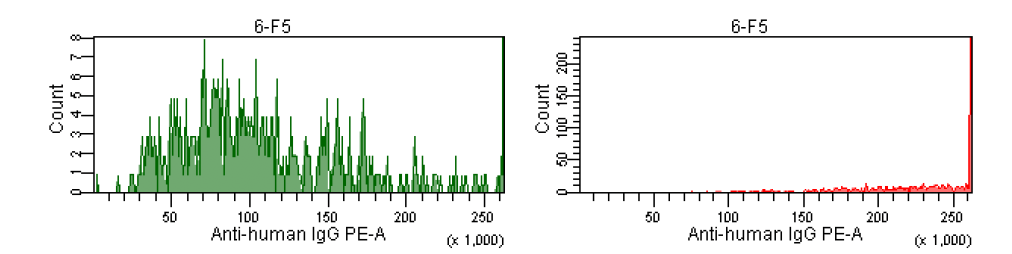


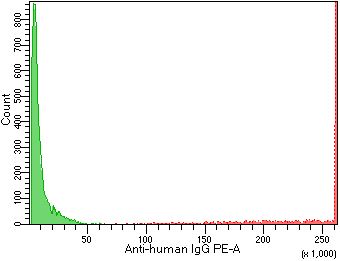


3C: Day of transplant, negative T (left, green) and B (right, red) cell FXCM


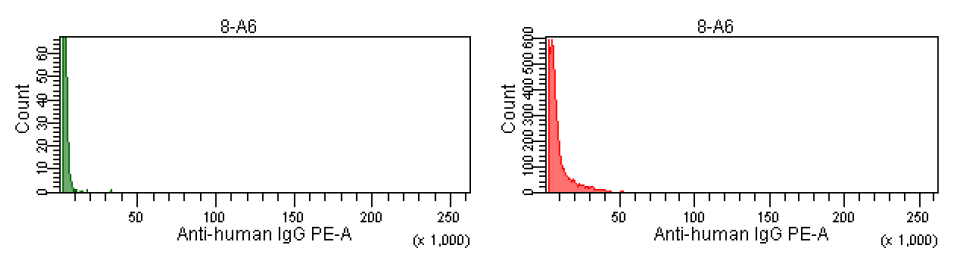


3D: Day 44 post-transplant, negative T (left, green) and B (right, red) cell FXCM
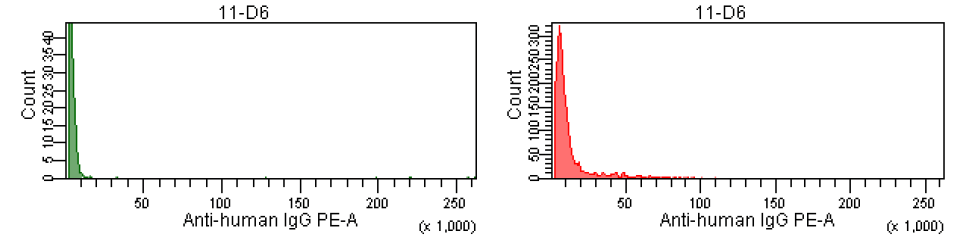


3E: Day 103 post-transplant, negative T (left, green) and positive B (right, red) cell FXCM


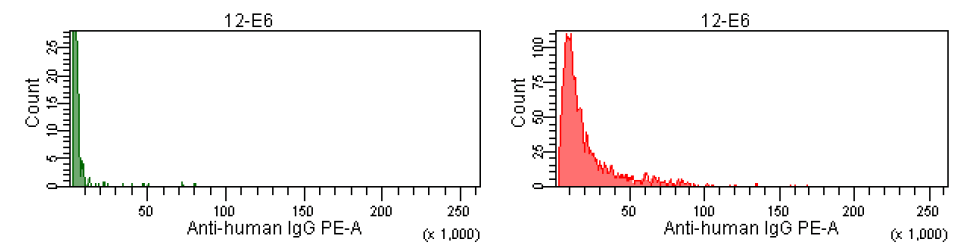


3F: Day 122 post-transplant, negative T (left, green) and B (right, red) cell FXCM


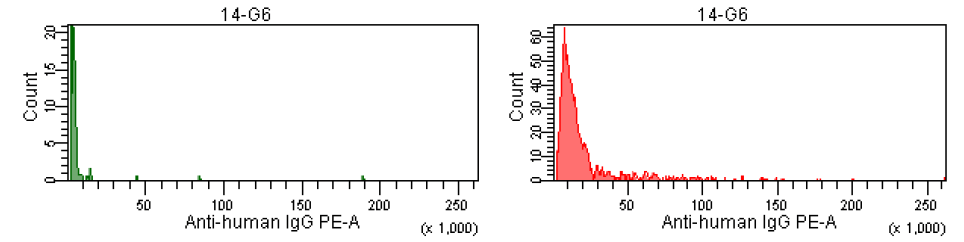


3G: Overlay of figures 3C and 3E showing the positive B cell FXCM result from 3E in yellow compared to the negative B cell FXCM result from 3C (day of Tx) in green. Note that at our center a linear readout is used that enables much higher sensitivity compared to traditional log-based readouts.


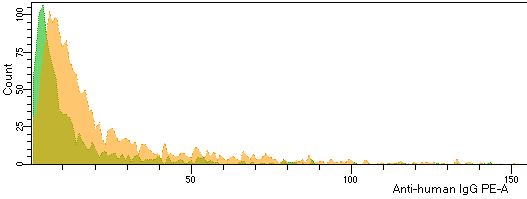

Supplement: Supplementary file 1 [file Supplementaryfile1.docx]
